# Effects of Exercise Training during Advanced Maternal Age on the Cognitive Function of Offspring

**DOI:** 10.3390/ijms23105517

**Published:** 2022-05-15

**Authors:** Tae-Woon Kim, Sang-Seo Park, Hye-Sang Park

**Affiliations:** 1 Department of Human Health Care, Gyeongsang National University, Jinju 52725, Korea; twkim0806@gnu.ac.kr; 2 School of Health and Kinesiology, University of Nebraska at Omaha, Omaha, NE 68182, USA; sangseopark@unomaha.edu; 3 Department of Physiology, College of Medicine, KyungHee University, Seoul 02453, Korea

**Keywords:** advanced maternal age, offspring, exercise, cognitive function, hippocampus, neuroplasticity, mitochondria

## Abstract

Advanced maternal age (AMA) denotes an age of ≥35 years during the time of delivery. Maternal metabolism affects the offspring’s physical and neurological development as well as their cognitive function. This study aimed to elucidate the effects of exercise training among old female animals on the cognitive function, hippocampal neuroplasticity, mitochondrial function, and apoptosis in the offspring. We found that the offspring of mothers with AMA without exercise training had decreased spatial learning and memory, brain-derived neurotrophic factor (BDNF) and postsynaptic density protein 95 (PSD-95) protein levels, neurogenesis, and mitochondrial function, as well as hippocampal cell death. Contrastingly, offspring of mothers with AMA with exercise training showed improved spatial learning, memory, hippocampal neuroplasticity, and mitochondrial function. These findings indicate that despite the AMA, increasing fitness through exercise significantly contributes to a positive prenatal environment for fetuses. The maternal exercises augmented the hippocampal levels of BDNF, which prevents decreased cognitive function in the offspring of mothers with AMA.

## 1. Introduction

Advanced maternal age (AMA) denotes an age of ≥35 years during the time of delivery. Considering the active economic participation of women, there has been an increase in the percentage of pregnancies during late marriage [1]. There are age-group differences in the characteristics of this trend, with younger generations showing an older age at childbirth [1]. Accordingly, considering the increase in the number of mothers with AMA, there has been increasing interest in high-risk pregnancies.

The risk associated with childbirth is positively correlated with maternal age. Pregnant women aged 35 years are more likely to develop hypertension and gestational diabetes than young women [2], and older mothers ≥45 years were related to risk for postpartum hemorrhage, premature deliveries, and maternal complications including cesarean section, preeclampsia, and placenta previa [3,4].

Furthermore, maternal female aging is negatively associated with the chance of pregnancy and the birth of a healthy baby. The relationship between maternal age and the offspring’s health results from the inheritance of mitochondrial extra-genomic material. Since this material degenerates upon aging, the mother with AMA has been described as a ‘genetic disease’ [5]. Childbirth problems among mothers with AMA include reduced maternal reproductivity, pregnancy-related conditions such as hypertension and diabetes, and an increased chance of birth defects related to abnormal chromosomes in aged eggs [2]. However, some studies have reported that maternal age is not associated with fetal mutations, birth defects [6], low birth weight, or premature birth [7]. Instead, compared with mothers with AMA, socioeconomic factors, including the parents’ education, occupation, and wealth, have stronger effects on childbirth outcomes. This suggests that various AMA-related factors, rather than AMA itself, are responsible for decreased birth weight.

Maternal metabolism affects the offspring’s physical and neurological development as well as cognitive function [8,9]. Maternal obesity and metabolic dysfunction can damage the hippocampus in the offspring, which is crucial for the learning and memory functions of the offspring [10]; moreover, they can suppress hippocampal brain-derived neurotrophic factor (BDNF) production as well as impair cognitive function and spatial learning in the offspring [11,12]. Therefore, it is important to improve the metabolism of mothers with AMA.

Physical activities increase metabolism [13] and aid in maintaining the physiological balance of neural cells in aged individuals [14]. Maternal exercise before and during pregnancy enhances metabolism and insulin sensitivity of the offspring [15]. Additionally, it improves their cognitive function, habitual behavior, and spatial learning, as well as increases the BDNF levels and the number of cells during hippocampus formation [16,17,18].

However, previous studies have only discussed the status of mothers with AMA, and related interventions from the perspective of maternal control before and after pregnancy as well as with the perspective that maternal age can affect fetuses within their prenatal environment [19,20]. Therefore, this study aimed to evaluate the effects of exercise training of mothers with AMA on hippocampal neuroplasticity, mitochondrial function, and cognition in offspring of mothers with AMA.

## 2. Results

### 2.1. Effects of Exercise in Mothers with AMA on Cognitive Function in Offspring

**Figure 1:**
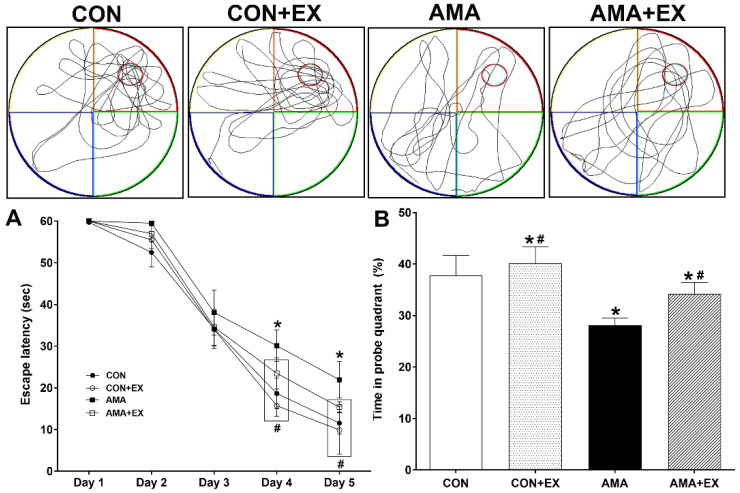
Effects of maternal exercise on spatial learning (**A**) and memory (**B**) in the offspring from mothers with advanced maternal age (AMA). The Morris water maze task was used to evaluate spatial learning and memory. CON: offspring from the control group; CON + EX: offspring from the control and exercised group; AMA: offspring from the advanced maternal age group; AMA + EX: offspring from the advanced maternal age and exercised group. Data are expressed as means ± standard error of the mean (SEM). * *p <* 0.05 compared to the CON group. # *p <* 0.05 compared to the AMA group.

The Morris water maze test was used to assess spatial learning and memory (Figure 1). Spatial learning was evaluated as the time until the animal finds the platform. In spatial learning ability, the AMA group took longer to visit the platform from days 4 and 5 compared to the CON group (*p* < 0.001) (Figure 1A), and no differences were observed among the other groups. For the spatial memory test at 24 h after 5 days of training, the AMA group showed significantly reduced spatial memory compared with the CON group (*p* = 0.03); however, the offspring of AMA with exercise training showed increased spatial memory (*p* = 0.034) (Figure 1B). Considered together, mothers with AMA had decreased spatial learning and memory in their offspring; however, mothers with AMA with exercise prevented decreased spatial learning and memory in offspring.

### 2.2. Effect of Exercise in Mothers with AMA on Hippocampal BDNF and PSD-95 Levels in the Offspring

**Figure 2:**
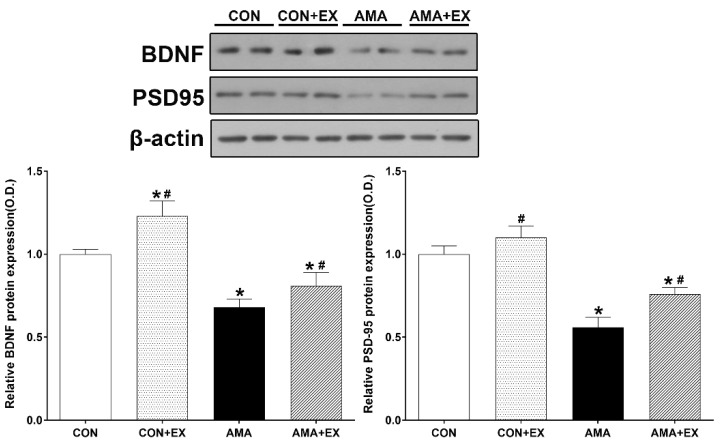
Effects of exercise on BDNF and PSD95 in the hippocampus of the offspring from mothers with advanced maternal age (AMA). CON: offspring from the control group; CON + EX: offspring from the control and exercised group; AMA: offspring from the advanced maternal age group; AMA + EX: offspring from the advanced maternal age and exercised group. Data are expressed as means ± standard error of the mean (SEM). * *p <* 0.05 compared to the CON group. # *p <* 0.05 compared to the AMA group.

We performed Western blotting changes in hippocampal BDNF and PSD-95 protein levels (Figure 2). For between-group comparisons, the value of the CON group was set to 1, and relative values were assigned to the other groups. Compared with the CON group, the AMA group showed significantly decreased BDNF (*p* < 0.001) and PSD-95 (*p* < 0.001) levels. Contrastingly, the AMA + EX group showed significantly increased BDNF (*p* < 0.001) and PSD-95 (*p* < 0.001) levels. Considered together, our results show decreased hippocampal BDNF and PSD-95 levels in the offspring from mothers with AMA; however, mothers with AMA with exercise prevented BDNF and PSD-95 inhibition in the hippocampus of the offspring.

### 2.3. Effect of Exercise in Mothers with AMA on Mitochondrial Ca^2+^ Retention and H_2_O_2_ Emission in the Hippocampus of Offspring

**Figure 3:**
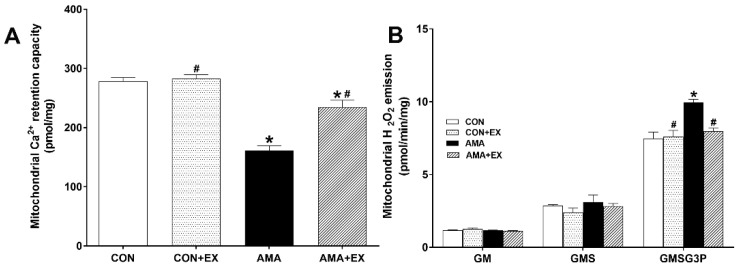
Effects of maternal exercise on mitochondrial Ca^2+^ retention (**A**) and H_2_O_2_ emission (**B**) in the hippocampus of offspring from mothers with advanced maternal age (AMA). CON: offspring from the control group; CON + EX: offspring from the control and exercised group; AMA: offspring from the advanced maternal age group; AMA + EX: offspring from the advanced maternal age and exercised group; GM: glutamate + malate; GMS: GM + succinate; GMSG3P: GMS + glycerol-3-phosphate. Data are expressed as means ± standard error of the mean (SEM). * *p <* 0.05 compared to the CON group. # *p <* 0.05 compared to the AMA group.

Compared with the CON group, the AMA group showed decreased mitochondrial Ca^2+^ retention capacity in the hippocampus (*p* < 0.001). Contrastingly, the AMA + EX group showed increased mitochondrial Ca^2+^ retention capacity (Figure 3A). The mitochondrial H_2_O_2_ emission rate was calculated using the Complex I substrate (glutamate + malate, GM), Complex 2 substrate (succinate, GMS), and the lipid substrate (glycerol-3 phosphate, GMSG3P). Compared with the CON group, the AMA group showed a significantly increased mitochondrial H_2_O_2_ emission rate in the hippocampus (GMSG3P: *p* < 0.001). Contrastingly, it was significantly decreased in the AMA + EX group (GMSG3P: *p* = 0.003) (Figure 3B). Considered together, decreased mitochondrial function was observed in the offspring of mothers with AMA, which was prevented by exercise. Therefore, prenatal exercise could effectively improve hippocampal mitochondrial function in the offspring by maintaining mitochondrial Ca^2+^ homeostasis and reducing the levels of reactive oxygen species.

### 2.4. Effect of Exercise in Mothers with AMA on Apoptosis and Cell Death in the Hippocampus of the Offspring

**Figure 4:**
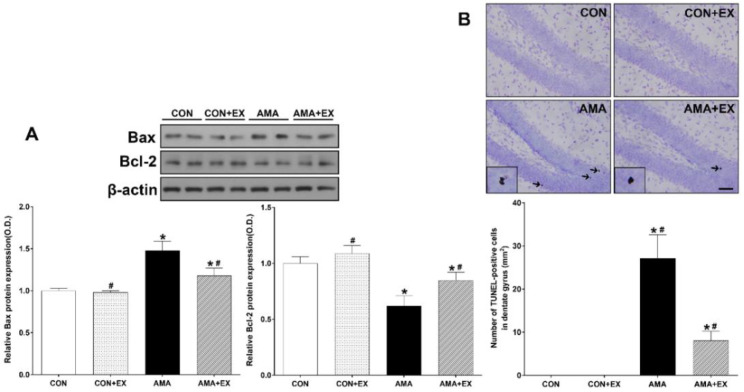
Effects of exercise on apoptosis (**A**) and cell death (**B**) in the hippocampus and dentate gyrus of offspring from mothers with advanced maternal age (AMA). Representative photomicrographs and data for TUNEL-positive cells are shown. The scale bar represents 50 µm. CON: offspring from the control group; CON + EX: offspring from the control and exercised group; AMA: offspring from the advanced maternal age group; AMA + EX: offspring from the advanced maternal age and exercised group. Data are expressed as means ± standard error of the mean (SEM). * *p <* 0.05 compared to the CON group. # *p <* 0.05 compared to the AMA group.

We analyzed Bax and Bcl-2 protein expressions to examine the changes in the protein levels (Figure 4). Additionally, we analyzed TUNEL-positive cells to examine hippocampal cell death. For between-group comparisons of cell death, the value of the CON group was set to 1, and the other groups were assigned relative values. Compared with the CON group, the AMA group showed increased Bax (*p* < 0.001) and decreased Bcl-2 protein expression (*p* < 0.001). Contrastingly, there was a significant decrease and increase in Bax (*p* < 0.001) and Bcl-2 (*p* < 0.001) protein expression in the AMA + EX group (Figure 4A). Compared with the CON group, the AMA group showed significantly increased TUNEL-positive cells in the hippocampus (*p* < 0.001). Contrastingly, the AMA + EX group showed decreased TUNEL-positive cells (*p* = 0.03) (Figure 4B). Considered together, increased hippocampal apoptosis and cell death were observed in the offspring from mothers with AMA, which was prevented by prenatal exercise.

### 2.5. Effect of Exercise in Mothers with AMA on Cell Differentiation and Neurogenesis in the Hippocampus of Offspring

**Figure 5:**
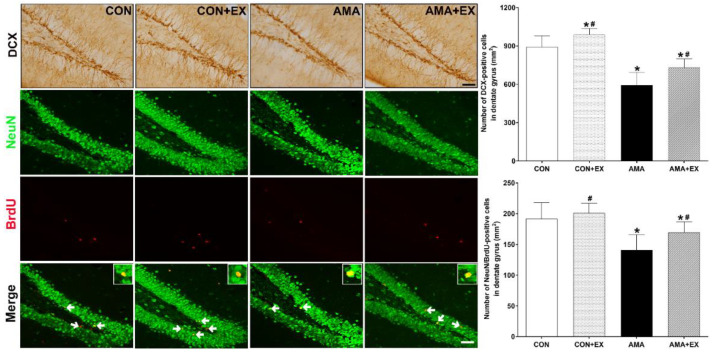
Effects of exercise on cell differentiation and neurogenesis in the hippocampal dentate gyrus of the offspring from mothers with advanced maternal age (AMA). Representative photomicrographs and data for DCX- and NeuN/BrdU-positive cells are shown. The scale bar represents 50 µm. CON: offspring from the control group; CON + EX: offspring from the control and exercised group; AMA: offspring from the advanced maternal age group; AMA + EX: offspring from the advanced maternal age and exercised group; NeuN: neuronal nuclear protein; BrdU: 5-bromo-2’-deoxyuridine. Data are expressed as means ± standard error of the mean (SEM). * *p <* 0.05 compared to the CON group. # *p <* 0.05 compared to the AMA group.

In order to examine hippocampal cell differentiation and neurogenesis, we analyzed DCX-positive cells and NeuN/BrdU-positive cells (Figure 5). Compared with the CON group, the AMA group showed significantly decreased DCX-positive cells (*p* = 0.04) and NeuN/BrdU-positive cells (*p* = 0.025) in the hippocampus. Contrastingly, they were significantly increased in the AMA + EX group (DCX-positive cells: *p* = 0.032; NeuN/BrdU-positive cells: *p* = 0.037). Considered together, the offspring from the mother with AMA showed decreased hippocampal cell differentiation and neurogenesis, which was prevented by prenatal exercise.

## 3. Discussion

Women’s fertility decreases upon aging; moreover, maternal age can affect the fetus within the prenatal environment through age-related changes [19,20]. As a more proactive measure to cope with childbirth in females with AMA, we administered exercise training to mouse mothers with AMA in order to improve their metabolism, and we examined its effect on hippocampal neuroplasticity, mitochondria function, and the cognitive function of their offspring. Mothers with AMA negatively affect the health of the offspring owing to physiological processes related to maternal age, including deterioration of the reproductive system as well as decreased quality of placenta or oocytes [21]. Previous studies reported that advanced parental age decreased intelligence and neurocognitive development as well as increased the risk of bipolar disorder and autism in offspring [22,23,24,25]. Notably, AMA is associated with low IQ scores in the offspring [19]. A Swiss population-based study reported decreased cognitive function in the offspring of mothers aged ≥30 years [26]. Animal studies have reported that the offspring of mothers with AMA have impaired learning and memory [27,28,29]. Consistent with previous findings, we observed decreased spatial learning, memory, and long-term memory in the offspring of mothers with AMA. Such problems of cognitive function are closely associated with the hippocampus. Prenatal or early postnatal adverse conditions exert long-term effects on *Arc*, *Egr1*, and *Fos* gene expression, which encode important transcription factors of hippocampal synaptic plasticity and connectivity [30,31,32]. Similarly, an AMA-affected prenatal environment may provide insufficient support for fetuses and can alter the developmental programming of the expression of various genetic factors in the hippocampus [33]. Han et al. [28] reported decreased cognitive function and hippocampal BDNF levels in the offspring of mothers with AMA. Additionally, they reported decreased nestin, doublecortin (DCX), and polysialylated neuronal cell adhesion molecule protein expression, which suggests that AMA can suppress hippocampal cell proliferation, survival, and migration [34]. Moreover, AMA affects hippocampal synapses by reducing PSD-93 expression [35]. The mitochondrial respiration function is gradually decreased in the fetus of females with AMA; furthermore, energy deficiency and subsequent secondary effects occur in the oocytes and fetal development [36,37]. Specifically, mitochondrial DNA is generally inherited from the mother, and maternal age affects mitochondrial genes [36]. We observed decreased BDNF, PSD-95, and DCX (cell proliferation) expression, decreased neurogenesis, and increased cell death in the hippocampus of offspring from mothers with AMA. Additionally, we observed decreased Ca^2+^ retention and increased H_2_O_2_ emission in the mitochondria of the hippocampus.

Exercise has positive effects on brain function. Specifically, it increases the expression of neurotrophic factors and genes [38]; in addition, it improves cognitive function and spatial learning by regulating hippocampal neurogenesis, synaptic plasticity, and learning [39].

Clinical evidence exists that prenatal exercise improves fetal health and early-childhood cognitive behavior [40,41,42]. Further, animal studies have shown that prenatal exercise in obese mothers enhanced the cognitive function of their offspring by increasing BDNF levels and neurogenesis as well as suppressing cell death in the hippocampus of their offspring [43]. In our study, compared with the AMA group, the AMA + EX group showed improved spatial learning and memory as well as increased hippocampal BDNF expression of their offspring.

A previous study demonstrated that maternal behavior and physical activities affected the expression of growth factors and transiently stimulated hippocampal development in the offspring [44]. Maternal exercise before and during pregnancy improves metabolism and insulin sensitivity in the offspring [15]. Furthermore, it improves the cognitive function of the offspring as well as BDNF levels and the number of cells during hippocampus formation [16,17,18]. BDNF is the most essential factor in neuroprotection. It improves cognitive function and inhibits cell death by promoting neural survival and cell differentiation [26,45], and it prevents neuronal apoptosis by inducing anti-apoptotic protein, such as Bcl-2, and inhibiting pro-apoptotic proteins, such as Bax [46]. Moreover, it is critically involved in mitochondrial metabolism by increasing respiratory coupling and adenosine triphosphate (ATP) production [47,48]. The maternal supply of BDNF affects fetal development [49]. We found that exercise in AMA increased PDS-95, neurogenesis, and cell differentiation as well as decreased cell death in the hippocampus of the offspring. Moreover, it improved the hippocampal mitochondrial function of the offspring, which was indicated by increased Ca^2+^ retention as well as decreased H_2_O_2_ emission.

In conclusion, the impact of maternal birth at an older age on offspring remains controversial. Even for AMA, fitness enhancement through exercise could play a crucial role in providing a positive prenatal environment for the fetus. Maternal exercise augmented the hippocampal levels of BDNF, which prevents decreased cognitive function in the offspring of mothers with AMA.

## 4. Materials and Methods

### 4.1. Animals

Female mice (C57BL/6 mice) were randomly divided into one of the following groups (*n* = 5 for each group): young maternal group (7 weeks old), young maternal exercise group, advanced maternal age group (10 months), and advanced maternal age and exercise. Females were placed together with males (7-weeks-old) during the dark cycle for one week to permit mating. The offspring were assigned to 4 groups after delivery (*n* = 10 in each group): Male offspring from the control group (young maternal; CON), male offspring from the control and exercised group (young maternal exercise; CON + EX), male offspring from the advanced maternal age group (AMA), and male offspring from the advanced maternal age and exercised group (AMA + EX). All the tests used 4-week-old male offspring. BrdU (Sigma, St. Louis, MO, USA) was administered intraperitoneally (i.p.) at 100 mg/kg/day once daily for gestation days 14–18 to observe neurogenesis of the offspring.

### 4.2. Exercise Protocol

As per a previously described exercise protocol method [50], the exercise groups exercised on a treadmill designed for animal use once daily for six days per week for 8 consecutive weeks before mating and during pregnancy. A load of exercise consisted of running at an initial speed of 3 m/min for the first 5 min, 5 m/min for the next 5 min, and 8 m/min for the last 20 min with 0° inclination. Before beginning the exercise, exercise groups underwent adjustment training for one week, which resulted in adaptation time and release of stress from the treadmill exercise for a week. The animals in the non-exercise group remained on the treadmill for the same duration of time without running.

### 4.3. Preparation of the Tissue Samples

Mice were euthanized immediately after the behavior test. In order to prepare brain slices, the animals were completely anesthetized with a CO_2_ concentration of 40–60%, perfused transcardially with 50 mM phosphate-buffered saline (PBS), and then fixed using a freshly prepared solution of 4% paraformaldehyde in 100 mM phosphate buffer (pH 7.4). The brains were then removed, post-fixed in the same fixative overnight, and transferred to a 30% sucrose solution for cryoprotection. Coronal sections with 40 μm thickness were created using a freezing microtome (Leica, Nussloch, Germany). From each group of 10 animals, 5 were used for immunohistochemistry and immunofluorescence, and 5 for used for Western blotting and analyses of mitochondrial function. The hippocampi for Western blot analyses were immediately stored at −80 °C until use.

### 4.4. Behavioral Analysis

Morris Water Maze

The Morris water maze test was conducted to measure spatial learning and memory ability. The test animals were acclimated by swimming freely for 60 s in a pool without a platform one day before the start of training. The educational training was performed 3 times per day for 5 days with a platform. If the animal was unable to find the location of the platform within 60 s, the experimenter guided the animal to the platform. Then, the animal remained on the platform for 30 s. A probe trial was conducted 24 h after the training session; free swimming for 60 s without a platform was automatically evaluated by video tracking to determine whether memory of the previous platform was retained or not.

### 4.5. Immunohistochemistry

In order to visualize cell differentiation, immunohistochemistry was performed to detect dentate gyrus (DG), and doublecortin (DCX) in the DG region of the hippocampus. The sections were incubated in PBS for 10 min and then washed three times for 3 min in PBS. The sections were then incubated in 1% H_2_O_2_ for 15 to 30 min. Sections were obtained from each brain and incubated overnight with goat anti-DCX antibody (1:500; Santa Cruz Biotechnology, Dallas, TX, USA) followed by incubation with biotinylated goat secondary antibody (1:200; Vector Laboratories, Burlingame, VT, USA) for another 90 min and then washed and incubated in Vector Elite ABC Kit (1:100; Vector Laboratories). Antibody-biotin–avidin–peroxidase complexes were visualized using the 3,3, Diaminobenzidine (DAB) Substrate Kit (Vector Laboratories). The slides were air-dried overnight at room temperature, and the coverslips were mounted using Permount.

### 4.6. Immunofluorescence

As per the previously described method [51], NeuN/BrdU-positive cells in the DG were evaluated by immunofluorescence. Briefly, the brain sections were permeabilized by incubation in 0.5% Triton X-100 in PBS for 20 min, incubated in 50% formamide-2× standard saline citrate at 65 °C for 2 h, denatured in 2 N HCl at 37 °C for 30 min, and then rinsed twice in 100 mM sodium borate (pH 8.5). The sections were incubated overnight with rat anti-BrdU antibody (1:200; Abcam, Cambridge, UK) and mouse anti-NeuN antibody (1:200; Millipore, Temecula, CA, USA). The brain sections were then washed in PBS and incubated with appropriate secondary antibodies for 1 h. The secondary antibodies were anti-mouse IgG Alexa Fluor-488 and anti-rat IgG Alexa Fluor-560.

### 4.7. Quantification of the Histology

Quantification of immunohistochemistry and immunofluorescence for the hippocampus, spanning −3.00 to −3.72 mm from the bregma, was obtained from each brain. Images were captured using a BX51TF microscope (Olympus, Tokyo, Japan) for immunohistochemistry and an FV3000 confocal microscope (Olympus, Tokyo, Japan) for subsequent Z-section (1 µm) analysis for immunofluorescence. For positive cell counts, images were photographed, and quantified hippocampal DG fields that were 2 slices of the hippocampus were randomly chosen from each brain section. A total of two fields was manually analyzed per mouse by one blinded experimenter. From each group of 10 animals, 5 were used for immunohistochemistry and immunofluorescence, and two slices from each group was analyzed, resulting in a total of 10 slices. Quantification of immunohistochemistry and immunofluorescence was calculated by dividing the number of positive cells by the area of the DG.

### 4.8. TUNEL Staining

In order to visualize DNA fragmentation, TUNEL staining was performed using an In Situ Cell Death Detection Kit (Roche Diagnostics, Risch-Rotkreuz, Switzerland) according to the manufacturer’s protocol. Sections were post-fixed in ethanol–acetic acid (2:1), rinsed, incubated with proteinase K (100 mg/mL), and then rinsed again. Next, they were incubated in 3% H_2_O_2_, permeabilized with 0.5% Triton X-100, rinsed, and incubated in the TUNEL reaction mixture. The sections were rinsed and visualized using Converter-POD with 0.03% DAB, counterstained with Cresyl violet, and mounted onto gelatin-coated slides. The slides were air-dried overnight at room temperature. A coverslip was added using Permount mounting medium.

### 4.9. Western Blotting

Hippocampal tissues were homogenized on ice and lysed in lysis buffer containing 50 mM Tris–HCl (pH 7.5), 150 mM NaCl, 0.5% deoxycholic acid, 1% Nonidet P40, 0.1% sodium dodecyl sulfate, 1 mM PMSF, and leupeptin 100 mg/mL. The protein content was measured using a Colorimetric Protein Assay Kit (Bio-Rad, Hercules, CA, USA). Thirty micrograms of protein was separated on sodium dodecyl sulfate-polyacrylamide gels and transferred onto a nitrocellulose membrane, which was incubated with mouse β-actin (1:1000; Santa Cruz Biotechnology, CA, USA), Bax (1:1000; Cell Signaling, Danvers, MA, USA), Bcl-2 (1:1000; Santa Cruz Biotechnology, CA, USA), BDNF (1:1000; Alomone, Jerusalem, Israel), PSD95, 1:1000; Cell Signaling, Danvers, MA, USA), primary antibodies. Horseradish peroxidase-conjugated secondary anti-mouse antibodies were used for β-actin and Bcl-2; anti-rabbit conjugated secondary antibodies were used for, Bax, BDNF, and PSD95.

### 4.10. Mitochondrial Ca^2+^ Retention Capacity

As per the previously described method [52], the mitochondrial calcium retention capacity was tested to assess the susceptibility of the permeability transition pore (PTP) to opening. Briefly, after grinding the hippocampal tissue, overlaid traces of changes in fluorescence induced by Calcium Green-5 N were measured continuously (ΔF/min) at 37 °C during state 4 respiration using a Spex FluoroMax 4 spectrofluorometer (Horiba Scientific, Edison, NJ, USA). After establishing the background ΔF (hippocampal tissue in the presence of 1 µM Calcium Green-5 N, 1 U/mL hexokinase, 0.04 mM EGTA, 1.5 nM thapsigargin, 5 mM 2-deoxyglucose, 5 mM glutamate, 5 mM succinate, and 2 mM malate), the reaction was initiated by Ca^2+^ pulses (12.5 nM), with excitation and emission wavelengths set to 506 and 532 nm, respectively. The total mitochondrial Ca^2+^ retention capacity prior to PTP opening (i.e., the release of Ca^2+^) is expressed in units of pmol/mg.

### 4.11. Mitochondrial H_2_O_2_ Emission

H_2_O_2_ emission was measured at 37 °C (ΔF/min) during state 4 respiration (10 μg/mL oligomycin) by continuously monitoring the oxidation of Amplex Red (excitation/emission λ = 563/587 nm) using a Spex FluoroMax 4 spectrofluorometer with 10 μM Amplex Red, 1 U/mL horseradish peroxidase, 10 μg/mL oligomycin, 1 mM malate + 2 mM glutamate (complex I substrates), 3 mM succinate (complex II substrate), and 10 mM glycerol-3-phosphate (lipid substrate). The H_2_O_2_ emission rate after subtracting the background value from the standard values (standard curve) was calculated from the ΔF/min gradient values, and the results were expressed in units of pmol/min/mg tissue weight.

### 4.12. Statistical Analyses

Cell counting and optical density quantification were performed using Image-Pro Plus (Media Cyberbetics Inc., Rockville, MD, USA) attached to a light microscope (Olympus). The data were analyzed using a one-way analysis of variance, followed by Tukey post hoc tests. All the values are expressed as means ± standard error of the mean, and *p*-values < 0.05 were considered significant.

## Data Availability

Data presented in this paper are available upon request to the correspondence.
